# Genome-wide analysis of autophagy-related genes in *Medicago truncatula* highlights their roles in seed development and response to drought stress

**DOI:** 10.1038/s41598-021-02239-6

**Published:** 2021-11-25

**Authors:** Mingkang Yang, Liping Wang, Chumin Chen, Xu Guo, Chuanglie Lin, Wei Huang, Liang Chen

**Affiliations:** State Key Laboratory for Conservation and Utilization of Subtropical Agro-bioresources, College of Life Sciences, South China Agricultural University, Guangzhou, 510642 China

**Keywords:** Plant genetics, Plant stress responses

## Abstract

Autophagy is a highly conserved process of degradation of cytoplasmic constituents in eukaryotes. It is involved in the growth and development of plants, as well as in biotic and abiotic stress response. Although autophagy-related (ATG) genes have been identified and characterized in many plant species, little is known about this process in *Medicago truncatula*. In this study, 39 *ATGs* were identified, and their gene structures and conserved domains were systematically characterized in *M. truncatula*. Many cis-elements, related to hormone and stress responsiveness, were identified in the promoters of *MtATGs*. Phylogenetic and interaction network analyses suggested that the function of *Mt*ATGs is evolutionarily conserved in *Arabidopsis* and *M. truncatula*. The expression of *MtATGs*, at varied levels, was detected in all examined tissues. In addition, most of the *MtATGs* were highly induced during seed development and drought stress, which indicates that autophagy plays an important role in seed development and responses to drought stress in *M. truncatula*. In conclusion, this study gives a comprehensive overview of *MtATGs* and provides important clues for further functional analysis of autophagy in *M. truncatula*.

## Introduction

Autophagy is an evolutionarily conserved degradation process in eukaryotes, which is involved in material and energy homeostasis through recycling of damaged cytoplasmic constituents and unwanted cellular materials^1^. In *Arabidopsis*, more than 30 autophagy-related genes (*ATGs*) have been identified via homology-based cloning using yeast *ATGs*^2,3^. They are involved in different stages of autophagosome formation, including phagophore induction, cargo capture, vesicle expansion and closure, and delivery of the vesicles to the vacuole^4^. ATGs are functionally classified into four core functional groups namely the ATG1 kinase complex, PI3K complex, ATG9 recycling complex, and two ubiquitin-like conjugation systems^5^. To date, *ATGs* have been characterized in many plant species including *Arabidopsis thaliana*, rice (*Oryza sativa*), maize (*Zea mays*), tobacco (*Nicotiana tabacum*), and wheat (*Triticum aestivum*)^6–9^.

Previous studies have indicated that autophagy is broadly involved in the growth and development of plants. It has been reported that autophagy-defective mutants show accelerated leaf senescence in *Arabidopsis*^10^. The *Osatg7* mutant showed complete sporophytic male sterility and reduced pollen germination activity, which suggests that autophagy plays critical roles in pollen development in rice^11^. Increasing evidence highlights the crucial role of autophagy in starch and lipid metabolism in plants^12,13^. Moreover, autophagy, as a quality control mechanism, mediates the degradation of cellular components and contributes to cellular homeostasis, which is necessary for plants to survive various abiotic and biotic stresses, such as nutrient deficiencies and heat, hypoxia, salt, and drought stresses^14–18^.

*Medicago truncatula* is a model plant for genetic research on legumes that interact with rhizobia to develop nodules for nitrogen fixation^19–21^. Despite its agronomical importance, the production of *M. truncatula* is threatened by abiotic stresses including high salt and drought stresses^22^. To facilitate our understanding of the mechanism and function of autophagy in *M. truncatula*, it is necessary to first identify all the *MtATGs*. Based on the complete genome sequence of *M. truncatula*^23,24^, herein, we provide a comprehensive description of *MtATGs*, including their genome-wide identification, characterization, and expression analysis. The results of this study lay the foundation for future research on the molecular mechanism of autophagy in *M. truncatula.*

## Materials and methods

### Identification of *MtATGs*

The identification of putative *MtATGs* was conducted using a bidirectional BLAST analytical strategy, and was performed using the BLASTP program that is integrated into the BioEdit software. First, the protein sequences of published autophagy-related genes in *Arabidopsis* were used to search against *M. truncatula* proteome sequences (MedtrA17_4.0) with the E-value cutoff at 1 × e^−5^. Then, all output *M. truncatula* protein sequences were aligned back to *Arabidopsis* proteome sequences. Only the *M. truncatula* genes that shared the highest similarities to the *At*ATGs in the second BLAST analysis were considered putative *Mt*ATGs. To further verify that the candidate genes are indeed *MtATGs*, the protein domain architectures were analyzed in the Pfam database (http://pfam.xfam.org)^25^. The chemical features of the *Mt*ATG proteins, including their molecular weights and theoretical isoelectric points, were obtained using the online tool ExPASy (http://web.expasy.org/compute_pi/). Subcellular localization of *Mt*ATGs was predicted using the CELLO system (http://cello.life.nctu.edu.tw). The gene and protein structures of *Mt*ATGs were extracted from the annotation file of the *M. truncatula* genome (MedtrA17_4.0) and visualized with the integrating bioinformatic analysis toolkit Tbtools^26^.

### Chromosomal location and gene duplication analysis

*MtATGs* were mapped to the chromosomes based on their physical positions in the *M. truncatula* genome (MedtrA17_4.0). To investigate the synteny of related genome regions in *M. truncatula*, putative orthologous genes were identified using the BLASTP program, and the results were used to generate a synteny map with the MCScanX program^27^. The genome locations of *MtATGs* and the duplicated gene pairs were visualized using Tbtools^26^.

### Protein sequence alignment and analysis of the phylogenetic relationship

The phylogenetic analysis of *Mt*ATGs was performed using the MEGA7 software^28^. The amino acid sequences of *Mt*ATGs and *At*ATGs in different gene families were aligned independently using the ClustalW algorithm with the default parameters. An unrooted phylogenetic tree was constructed with the neighbor-joining statistical method, and the following parameters were used: uniform rates are used as rates among sites, gaps/missing data are treated as pairwise deletion, and the bootstrap analysis was performed with 1000 replicates to obtain a support value for each branch.

### Identification of cis-elements

The 1.5 kb genomic DNA sequence upstream of the initiation codon of each *MtATG* was retrieved from the *M. truncatula* genome (MedtrA17_4.0). The assumed cis-elements of MtATGs were predicted using the PlantCARE web servers (http://bioinformatics.psb.ugent.be/webtools/plantcare/html/)^29^.

### Construction of the protein–protein interaction (PPI) network

The PPI networks were constructed using the STRING database (http://www.string-db.org). A total of 39 MtATGs were selected as input, and the PPI network of the MtATGs was constructed with a medium confidence (0.4).

### Analysis of the expression profiles using microarray data

The *M. truncatula* microarray data were downloaded from the MtGEA v3 database (https://mtgea.noble.org/v3/)^30^. Expression values were normalized using the z-score method, and plotted using GraphPad Prism 8.

### Plant materials and growth conditions

*Medicago truncatula* (cv. Jemalong A17) seeds were scarified with sulfuric acid, and vernalized on wetted filter paper at 4 °C for 7 days. Seedlings were grown in a greenhouse at 24 °C, 16-h light/8-h dark cycle, with humidity ranging from 60 to 80%. Different plant tissues (roots, stems, leaves, petioles, buds, flowers, and pods) were harvested from multiple plants. Material for the seed developmental was collected from pods at 5 different stages. For drought stress, 7-day-old seedlings were treated by withholding watering for 2 days. For mannitol treatment, 2-weeks-old seedlings were transferred to liquid 1/2 MS medium supplemented with 300 mM mannitol for additional 2 days. All plant samples were frozen immediately in liquid nitrogen after harvest and stored at − 80 °C until use. Plant material collections in this study complied with relevant institutional, national, and international guidelines and legislation.

### RNA isolation and quantitative PCR (qPCR) analysis

Total RNA was extracted with TRIzol reagent (Invitrogen) according to the manufacturer’s instructions. The isolated RNA was reverse transcribed using ReverTra Ace qPCR RT Master Mix with gDNA Remover Kit (TOYOBO). qPCR was performed using the CFX Connect Real-Time PCR System (Bio-Rad) with the SYBR Premix ExTaq Mix (Takara). *MtACTIN* (Medtr2g008050) was used as a reference gene. Three technical replicates were used for each reaction. The gene-specific primers for the qPCR analysis are listed in Supplementary Table S4.

### Protein blotting analysis

Western blotting analysis of ATG8 lipidation was performed as previously described^31^. 2-weeks-old seedlings were ground in liquid nitrogen and homogenized in ice-cold RIPA buffer (50 mM Tris–HCl pH8.0, 150 mM NaCl, 1% NP-40, 0.5% Sodium Deoxycholate, 0.5% PvPP, 0.1% SDS). After centrifuged for 15 min at 12,000*g*, the supernatant fraction was transferred to a new microcentrifuge tube, and electrophoresis with 15% SDS-PAGE supplemented with 6 M Urea. Anti-ATG8a antibodies (ab77003, Abcam) were used in the immunoblotting analysis.

### Monodansylcadaverine (MDC) staining and microscopy

MDC staining was performed as previously described^32^. Briefly, lateral roots of *M. truncatula* were detached and stained with 0.75 mM MDC for 1 h. The root cells were observed using LSM 780 inverted microscope (Carl Zeiss) with a DAPI-specific filter.

## Results

### Genome-wide identification of *ATGs* in *M. truncatula*

To identify *Mt*ATGs, the BLASTP algorithm was employed in searches against *M. truncatula* proteome sequences (MedtrA17_4.0) using the amino acid sequences of *A. thaliana* ATGs (*At*ATGs) as queries. A total of 39 *Mt*ATGs were identified in *M. truncatula* (Table 1, Supplementary Tables S1, S2). The lengths of the *Mt*ATGs ranged from 62 amino acids to 3768 amino acids. Most of the *Mt*ATGs (*Mt*ATG2, *Mt*ATG3, *Mt*ATG4, *Mt*ATG5, *Mt*ATG6, *Mt*ATG7, *Mt*ATG10, *Mt*ATG11, *Mt*ATG12, *Mt*ATG101, *Mt*VPS15, and *Mt*VPS34) contained a single member. A few of them (*Mt*ATG1, *Mt*ATG8, *Mt*ATG9, *Mt*ATG13, *Mt*ATG16, and *Mt*ATG18) contained multiple members, ranging from two to eight in different groups (three in the *Mt*ATG1 family, eight in the *Mt*ATG8 family, two in the *Mt*ATG9 family, three in the *Mt*ATG13 family, three in the *Mt*ATG16 family, and eight in the *Mt*ATG18 family) (Table 1).

**Table 1:** Information related to *ATGs* and their encoded proteins in *Medicago truncatula*.

Gene name	Locus ID	Length (aa)	MW (kDa)	PI	Subcellular localization	Chromosome location
*MtATG1a*	Medtr8g024100	696	77.38	5.69	Nuclear	chr8:8817813..8824200
*MtATG1b*	Medtr4g019410	737	82.03	7.13	Nuclear	chr4:6057862..6065974
*MtATG1t*	Medtr3g095620	290	32.87	7.09	Extracellular	chr3:43689826..43692334
*MtATG2*	Medtr4g086370	1975	216.66	5.07	Nuclear	chr4:33827078..33844760
*MtATG3*	Medtr4g036265	310	35.27	4.5	Cytoplasmic	chr4:13052245..13057301
*MtATG4*	Medtr7g081230	487	53.82	5.04	Chloroplast	chr7:30993699..30998401
*MtATG5*	Medtr5g076920	361	41.14	4.31	Nuclear	chr5:32806624..32813118
*MtATG6*	Medtr3g018770	509	58.07	6.45	Nuclear	chr3:5165817..5174556
*MtATG7*	Medtr0003s0540	698	76.88	5.38	Plasma membrane	scaffold0003:305855..310747
*MtATG8a*	Medtr2g023430	120	13.72	9.3	Mitochondrial	chr2:8277496..8280062
*MtATG8b*	Medtr4g037225	120	14.13	7.82	Nuclear	chr4:13715664..13717673
*MtATG8c*	Medtr4g048510	120	13.89	9.29	Cytoplasmic	chr4:17207135..17210565
*MtATG8d*	Medtr2g088230	108	12.37	7.51	Cytoplasmic	chr2:37163050..37165680
*MtATG8e*	Medtr4g101090	122	14.06	8.76	Cytoplasmic	chr4:41752327..41755124
*MtATG8f*	Medtr1g086310	121	14.10	8.18	Cytoplasmic	chr1:38625116..38626309
*MtATG8g*	Medtr4g123760	118	13.82	9.74	Nuclear	chr4:51007802..51010377
*MtATG8h*	Medtr7g096540	62	7.09	9.1	Extracellular	chr7:38739985..38740615
*MtATG9a*	Medtr7g096680	893	103.32	6.56	Plasma membrane	chr7:38799346..38805558
*MtATG9b*	Medtr1g070160	866	99.95	6.7	Plasma membrane	chr1:30830518..30837261
*MtATG10*	Medtr8g010140	235	27.01	4.77	Extracellular	chr8:2577226..2579513
*MtATG11*	Medtr4g130370	1154	129.95	5.9	Nuclear	chr4:54307709..54314660
*MtATG12*	Medtr8g020500	124	10.59	9.07	Plasma membrane	chr8:7198686..7202464
*MtATG13a*	Medtr5g068710	584	65.58	9.42	Nuclear	chr5:29098584..29102600
*MtATG13b*	Medtr3g095570	633	70.29	8.72	Nuclear	chr3:43671041..43677624
*MtATG13c*	Medtr8g093050	583	65.62	8.73	Nuclear	chr8:38885014..38889871
*MtATG16a*	Medtr3g075400	509	55.88	6.65	Nuclear	chr3:34315394..34318708
*MtATG16b*	Medtr4g104380	514	56.74	6.21	Nuclear	chr4:43185561..43189052
*MtATG16c*	Medtr4g007500	364	40.66	4.66	Nuclear	chr4:1115999..1117649
*MtATG18a*	Medtr1g083230	385	42.67	7.83	Plasma membrane	chr1:37037962..37041428
*MtATG18b*	Medtr4g130190	372	40.38	7.64	Plasma membrane	chr4:54209571..54215694
*MtATG18c*	Medtr7g108520	418	45.73	7.44	Plasma membrane	chr7:44206217..44209925
*MtATG18d*	Medtr1g088855	354	39.70	9.2	Plasma membrane	chr1:39776324..39778721
*MtATG18e*	Medtr3g093590	415	46.14	7.95	Plasma membrane	chr3:42763022..42768303
*MtATG18f*	Medtr2g082770	901	98.20	6.87	Nuclear	chr2:34727900..34734357
*MtATG18g*	Medtr1g089110	967	105.32	6.11	Nuclear	chr1:40103141..40108943
*MtATG18h*	Medtr1g082300	913	99.63	5.95	Nuclear	chr1:36587909..36596198
*MtATG101*	Medtr8g079240	218	25.48	6.43	Cytoplasmic	chr8:33765931..33771318
*MtVPS15*	Medtr6g088835	1536	171.92	6.9	Plasma membrane	chr6:33989403..33999113
*MtVPS34*	Medtr5g034120	808	92.65	6.47	Cytoplasmic	chr5:14747697..14758501

**Figure 1:**
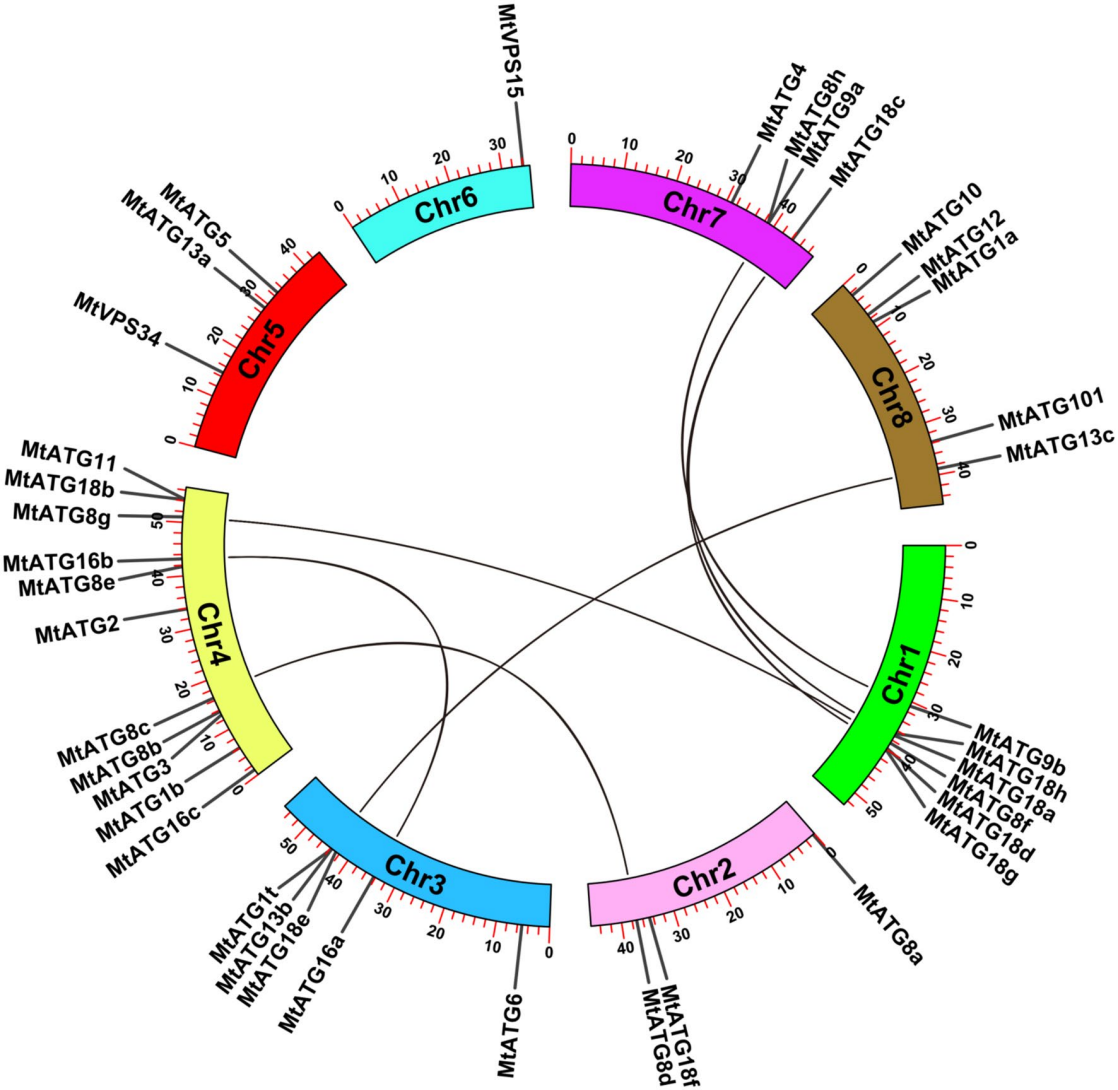
Chromosomal distribution and gene duplication of *MtATGs*. The genome locations of *MtATGs* were retrieved from the *M. truncatula* genome annotation (MedtrA17_4.0) except for MtATG7. The duplications between *MtATGs* were analyzed by the MCScanX program and linked with black lines.

The chromosomal distribution of *MtATGs* determined using the TBtools software is shown in Fig. 1. In total, 38 *MtATGs* were found to be distributed across all eight chromosomes except for *MtATG7*, which could not be mapped to any chromosome according to data from MedtrA17_4.0 (Fig. 1). The number of *MtATGs* located on each chromosome varies dramatically. Chromosome 4 (Chr4) contains the maximum number (11) of *MtATGs*, whereas chromosome 6 has only one *MtATG* gene. Gene duplication is important for adaptation of plants to adverse and complex environments. In *M. truncatula*, 7 pairs of *MtATGs* were predicted to be segmentally duplicated. As shown in Fig. 1, these 7 pairs of duplicated *MtATGs* (*MtATG8c* and *MtATG8d*, *MtATG8g* and *MtATG8f.*, *MtATG9a* and *MtATG9b*, *MtATG13b* and *MtATG13c*, *MtATG16a* and *MtATG16b*, *MtATG18a* and *MtATG18c*, *MtATG18d* and *MtATG18c*) are distributed across chromosomes 1, 2, 3, 4, 7, and 8. These duplications may have led to the expansion of *MtATG* families in *M. truncatula*.

The subcellular localization of the *Mt*ATGs was predicted using the CELLO system (http://cello.life.nctu.edu.tw). Most of the *Mt*ATGs were predicted to localize to the nucleus, plasma membrane, and cytoplasm, followed by extracellular space, chloroplast, and mitochondria (Table 1, Supplementary Figure S1). Furthermore, some *Mt*ATG families exhibited different subcellular localization. For example, *Mt*ATG8 proteins were predicted to be mainly cytoplasmic or nuclear, but were also found to localize to the mitochondria and extracellular space (Table 1). The prediction was the same for *Mt*ATG18 family members, which were localized to both the plasma membrane and nucleus (Table 1). Taken together, the diverse subcellular localization of *Mt*ATGs implies that they have distinct functions.

### Phylogenetic analysis of *Mt*ATGs

**Figure 2:**
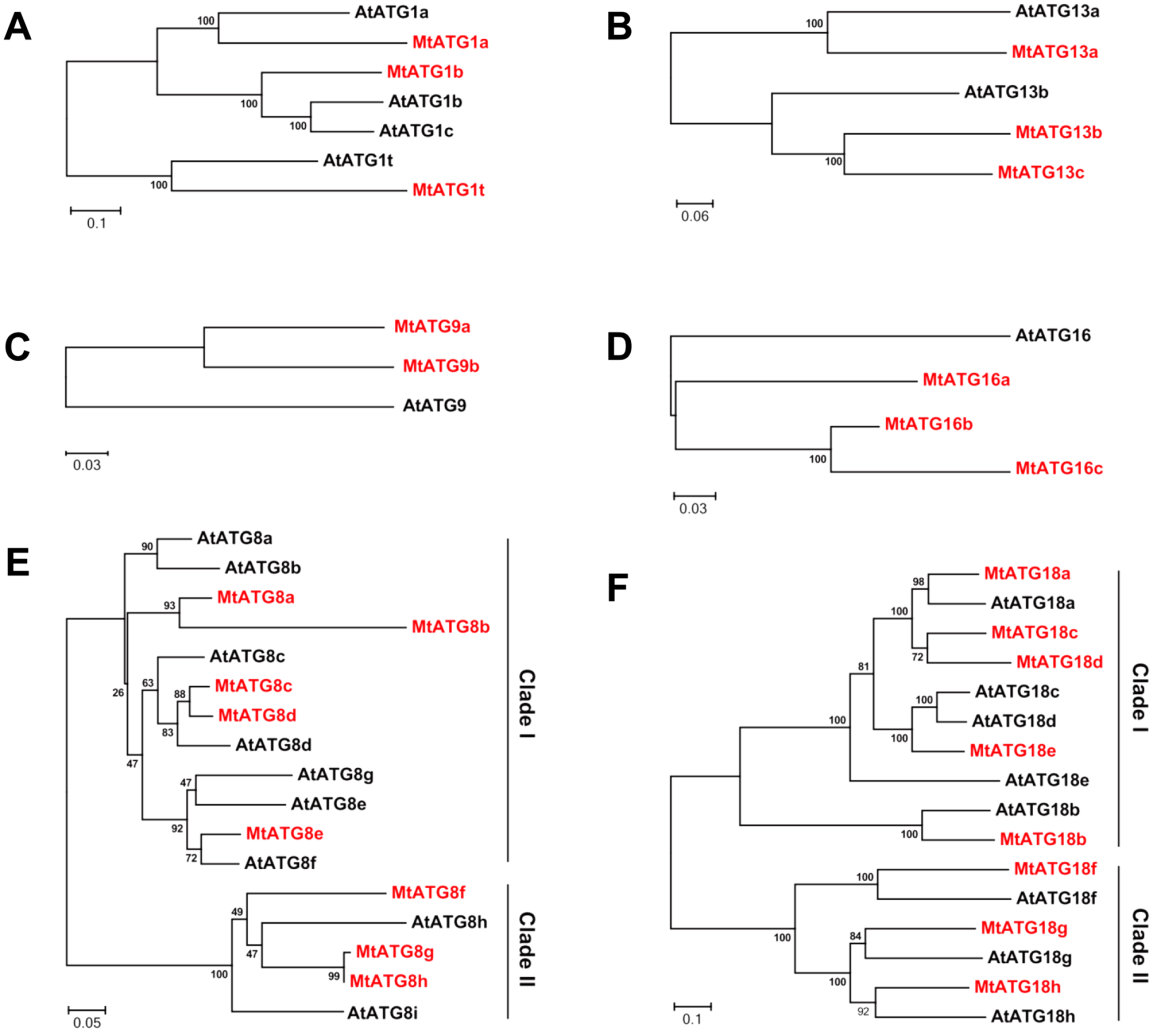
Phylogenetic analysis of ATGs from *Medicago truncatula* and *Arabidopsis thaliana*. Phylogenetic tree of ATG1 (**A**), ATG13 (**B**), ATG9 (**C**), ATG16 (**D**), ATG8 (**E**), and ATG18 (**F**) families in *M. truncatula* and *Arabidopsis*. The unrooted tree was constructed using MEGA7 based on the multiple sequence alignment of the ATG protein sequences by the neighbor-joining (NJ) method. The number at each node represents the bootstrap value from 1000 replicates.

To evaluate the evolutionary relationships of *Mt*ATGs, we conducted a phylogenetic analysis using the amino acid sequences of the multi-member subfamilies (*Mt*ATG1, *Mt*ATG8, *Mt*ATG9, *Mt*ATG13, *Mt*ATG16, and *Mt*ATG18) and their orthologs from *Arabidopsis*. As shown in Fig. 2, members of the *Mt*ATG1 and *Mt*ATG13 families were clustered in two branches (Fig. 2A,B). There are two ATG9s and three ATG16s in *M. truncatula*, whereas only one ATG9 and ATG16 in *Arabidopsis* (Fig. 2C,D). ATG8 plays a central role in autophagy by promoting autophagosome formation and cargo recruitment. As in *Arabidopsis*, eight *Mt*ATG8 members were clustered into two distinct groups in *M. truncatula*: *Mt*ATG8a, *Mt*ATG8b, *Mt*ATG8c, *Mt*ATG8d, and *Mt*ATG8e were grouped into clade I, whereas *Mt*ATG8f, *Mt*ATG8g, and *Mt*ATG8h were clustered in clade II (Fig. 2E). *Mt*ATG8 proteins showed high identity with ATG8 proteins from *Arabidopsis*, except for *Mt*ATG8h, in which half of the amino acids from the N-terminus were absent (Supplementary Figure S2). The C-terminal glycine residue in ATG8, which is exposed upon protease cleavage by ATG4, is essential for the conjugation of ATG8 to phosphatidylethanolamine^33^. However, *Mt*ATG8b did not contain the C-terminal glycine residue. This result indicates that *Mt*ATG8b might function in other biological processes independent of autophagy. In addition, one *Mt*ATG8 member of clade II, *Mt*ATG8f, had a C-terminal extension after the Gly residue, whereas the *At*ATG8 members of clade II lack the C-terminal extension (Supplementary Figure S2). Eight *Mt*ATG18 members were also clustered in two branches like the *Mt*ATG8 family members (Fig. 2F). Clade I of *Mt*ATG18 family consisted of *Mt*ATG18a, *Mt*ATG18b, *Mt*ATG18c *Mt*ATG18d, and *Mt*ATG18e, whereas clade II was made up of *Mt*ATG18f, *Mt*ATG18g, and *Mt*ATG18h (Fig. 2F).

### Analyses of gene structures and distribution of conserved domains

**Figure 3:**
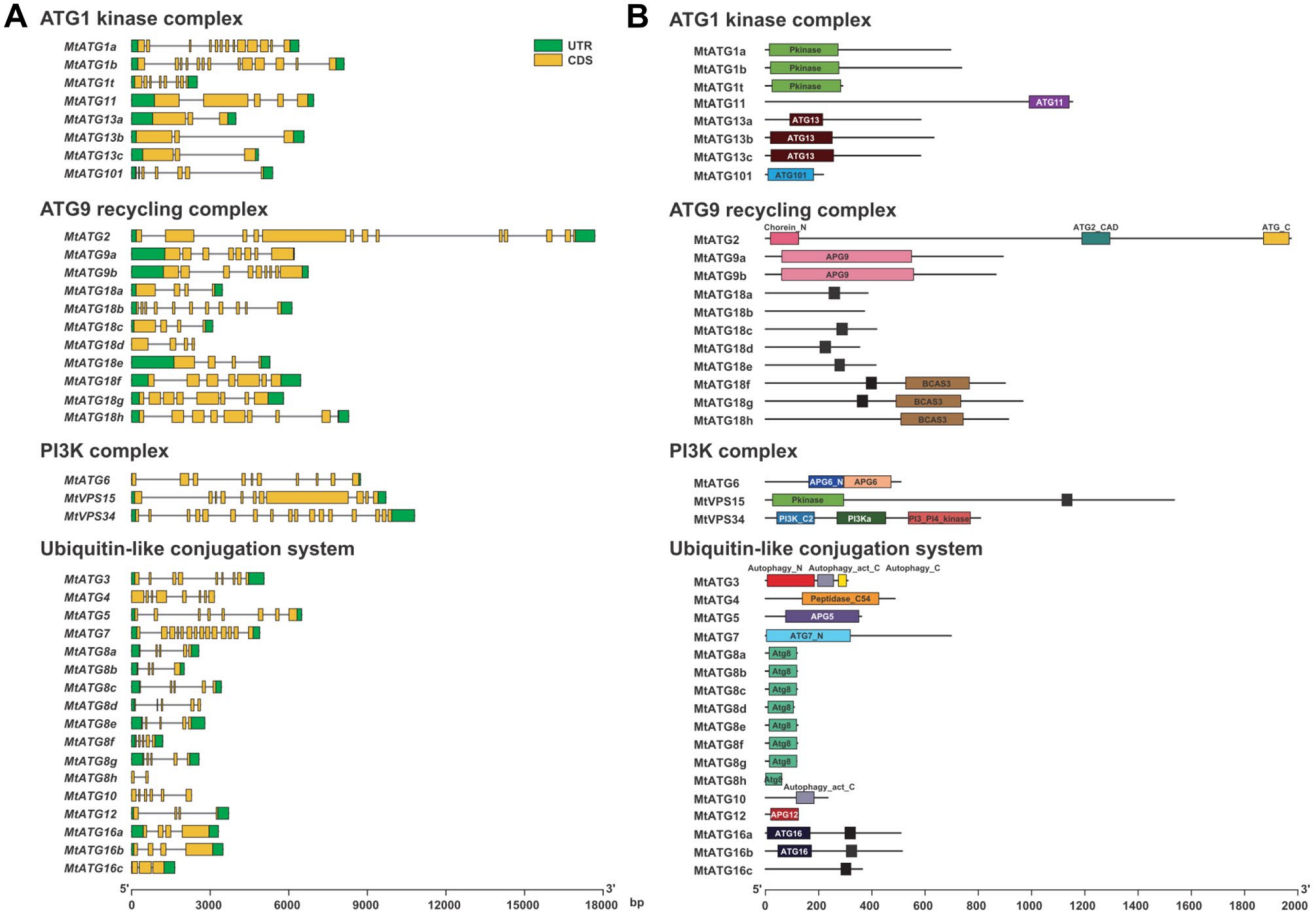
Gene structure and conserved domains of *Mt*ATGs. (**A**) Gene structure of *MtATGs* is illustrated according to *M. truncatula* genome annotation (MedtrA17_4.0), and the lengths of the exons and introns of each *MtATG* are exhibited proportionally. *MtATGs* are grouped based on their biological function in the autophagy pathway. (**B**) The domain architectures were predicted using the Pfam database, and protein lengths of the *Mt*ATGs were acquired from the *M. truncatula* genome annotation (MedtrA17_4.0). The black box represents the WD40 domain.

Gene structure is closely related to the expression pattern and function divergence of members of multigene families. Gene structure analysis revealed that all the *MtATGs* contain introns, with the number of exons ranging from 2 to 17 (Fig. 3A). In addition, similar exon–intron patterns and the same number of exons were observed in some *ATG* subfamilies, such as *MtATG1a/b*, *MtATG8a/c/d/e/f/g*, *MtATG13a/b/c*, *MtATG18a/c/d/e*, and *MtATG18g/h* (Fig. 3A). The similar gene structures suggest functional redundancy among these genes. However, differences in exon–intron patterns and exon numbers were also seen within some subfamilies, such as *MtATG1t*, *MtATG8b/h*, and *MtATG18b/f* (Fig. 3A).

The conserved domains of *Mt*ATGs were detected using the Pfam database^25^. In general, the composition of the conserved domains in *Mt*ATGs is comparable to that in *Arabidopsis*. Furthermore, members of the same *Mt*ATG families have similar domains. For example, all three *Mt*ATG1 proteins contain a protein kinase domain (Pkinase) at their N-terminus (Fig. 3B). In addition, almost all *Mt*ATG8 proteins (except *Mt*ATG8h) are similar in length and have identical ATG8 domains (Fig. 3). A similar phenomenon was also observed in the *Mt*ATG9 and *Mt*ATG13 subfamilies. However, exceptions were also found in the *Mt*ATG16 and *Mt*ATG18 subfamilies. All the *Mt*ATG16 family proteins have a C-terminal WD40 domain, but lack an N-terminal ATG16 domain in *Mt*ATG16c (Fig. 3B). *Mt*ATG18 proteins contain the WD40 domain except for *Mt*ATG18b and *Mt*ATG18h, but members of clade II (*Mt*ATG18f/g/h) have a C-terminal BCAS3 domain that is absent in members of clade I (Fig. 3B). The differences in the gene structure and conserved domains may be related to functional divergence among the different gene products within some *MtATG* families.

### Analysis of cis-elements in the promoter regions of *MtATGs*

**Figure 4:**
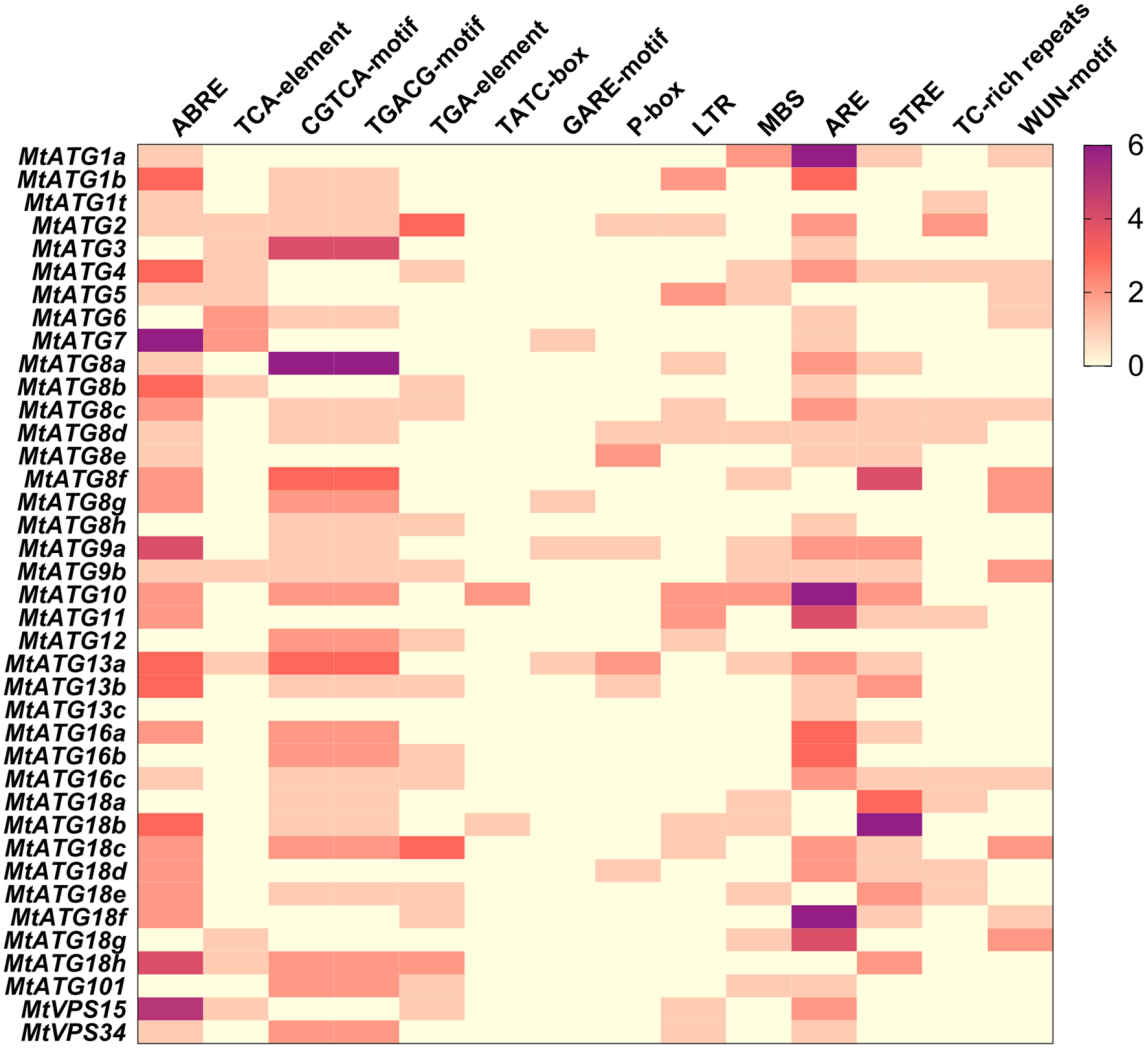
The number of cis-elements in promoters of *MtATGs*. The assumed cis-elements of *MtATGs* predicted using the PlantCARE web servers, and the number of cis-elements in each promoter of *MtATGs* are visualized using a heatmap generated with GraphPad Prism 8.

Cis-elements regulate genes through interactions with their corresponding transcription factors. To further understand the gene regulation network of *MtATGs*, cis-elements were identified using the online tool PlantCARE^29^. Ninety-two putative cis-elements were found among *MtATG* promoters (Supplementary Table S3). Among these, the TATA-box and CAAT-box are the most common cis-elements. Many of the identified cis-elements, such as ABRE (abscisic acid-related), TCA-element (salicylic acid-related), TCCACCT-motif and TGACG-motif (MeJA-related), TGA-element (auxin-related), TATC-box, and P-box and GARE-motif (gibberellin-related), are involved in hormone responsiveness (Fig. 4). Among these, cis-elements that respond to MeJA and ABA were found to be the most abundant. In addition, some stress-related elements are mainly related to anaerobic (ARE), defense (STRE and TC-rich repeats), drought (MBS), low temperature (LTR), and wound (WUN-motif) stresses (Fig. 4). The diversity of cis-elements in the promoter regions of *MtATGs* provided evidence for their potential biological functions in response to phytohormone, abiotic and biotic stresses.

### Analysis of the protein–protein interaction network of *Mt*ATGs

**Figure 5:**
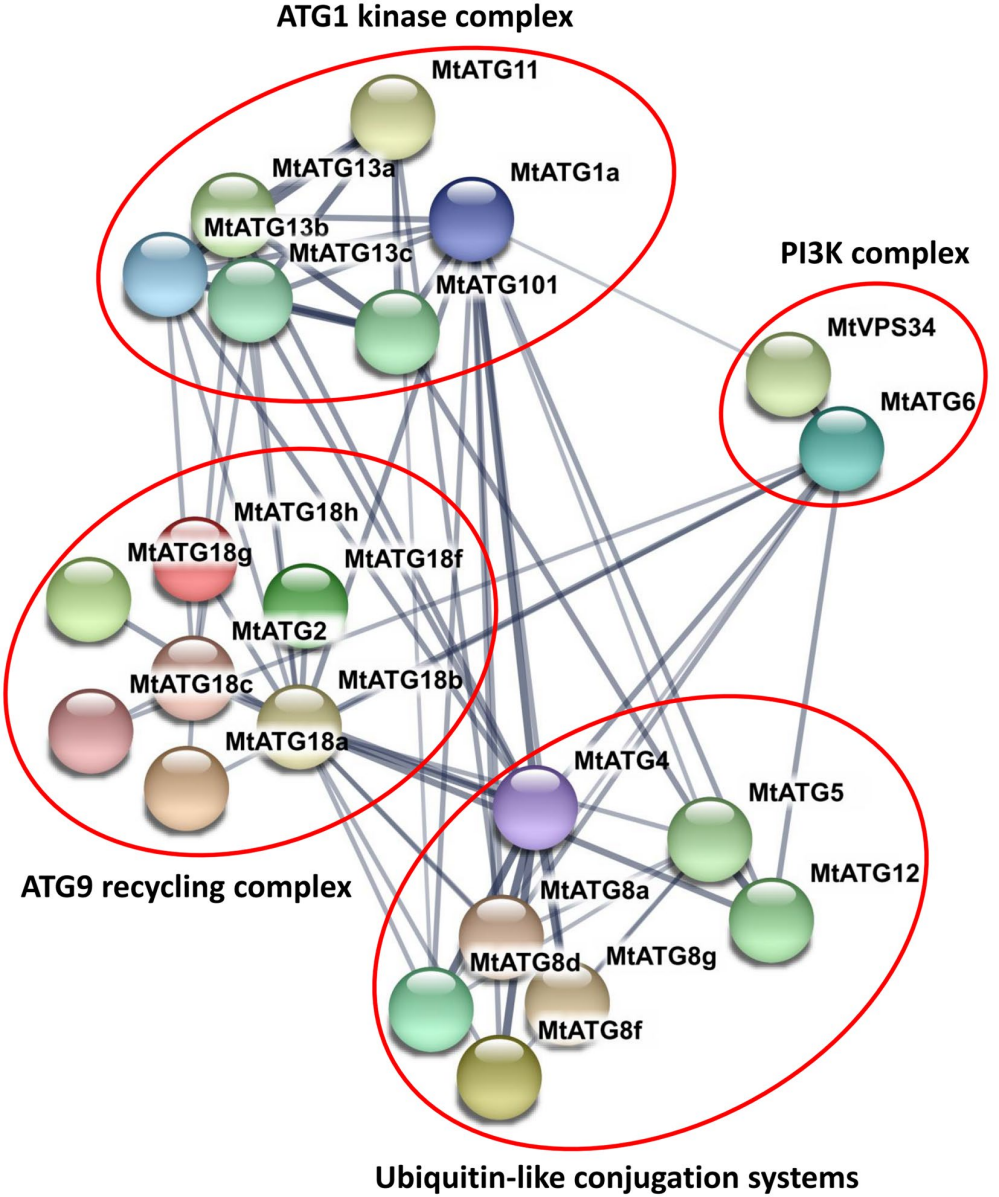
Protein–protein interaction network of *Mt*ATGs. The associations among proteins are derived from various channels: textmining, experiments, databases, coexpression, neighborhood, gene fusion, and co-occurrence. The thickness of the lines indicates the strength of data support.

To investigate the protein–protein interaction (PPI) between *Mt*ATGs, all the 39 *Mt*ATGs were submitted to the STRING (Search Tool for the Retrieval of Interacting Genes database) website. Twenty-two *Mt*ATGs were found to form a complex interaction network that can be divided into four major modules according to the functional classification in *Arabidopsis* (Fig. 5). In the first module, *Mt*ATG1a, *Mt*ATG11, *Mt*ATG101, and three *Mt*ATG13 members (*Mt*ATG13a, *Mt*ATG13b, *Mt*ATG13c) interact with each other and function as the ATG1 kinase complex. The second module consists of two members of the PI3K complex, *Mt*ATG6 and *Mt*VPS34. *Mt*ATG2 and six *Mt*ATG18 family members (*Mt*ATG18a, *Mt*ATG18b, *Mt*ATG18c, *Mt*ATG18f, *Mt*ATG18g, and *Mt*ATG18h), making up the third module, play a role in autophagic membrane recruitment. The last module, composed of *Mt*ATG4, *Mt*ATG5, *Mt*ATG12, and four *Mt*ATG8 members (*Mt*ATG8a, *Mt*ATG8d, *Mt*ATG8f, and *Mt*ATG8g), serves as the ubiquitin-like conjugation system. This interaction pattern of *Mt*ATGs is similar to that of *Arabidopsis,* suggesting that ATGs are possibly evolutionarily conserved in *Arabidopsis* and *M. truncatula*.

### Expression patterns of *MtATGs* in different tissues and during seed development

**Figure 6:**
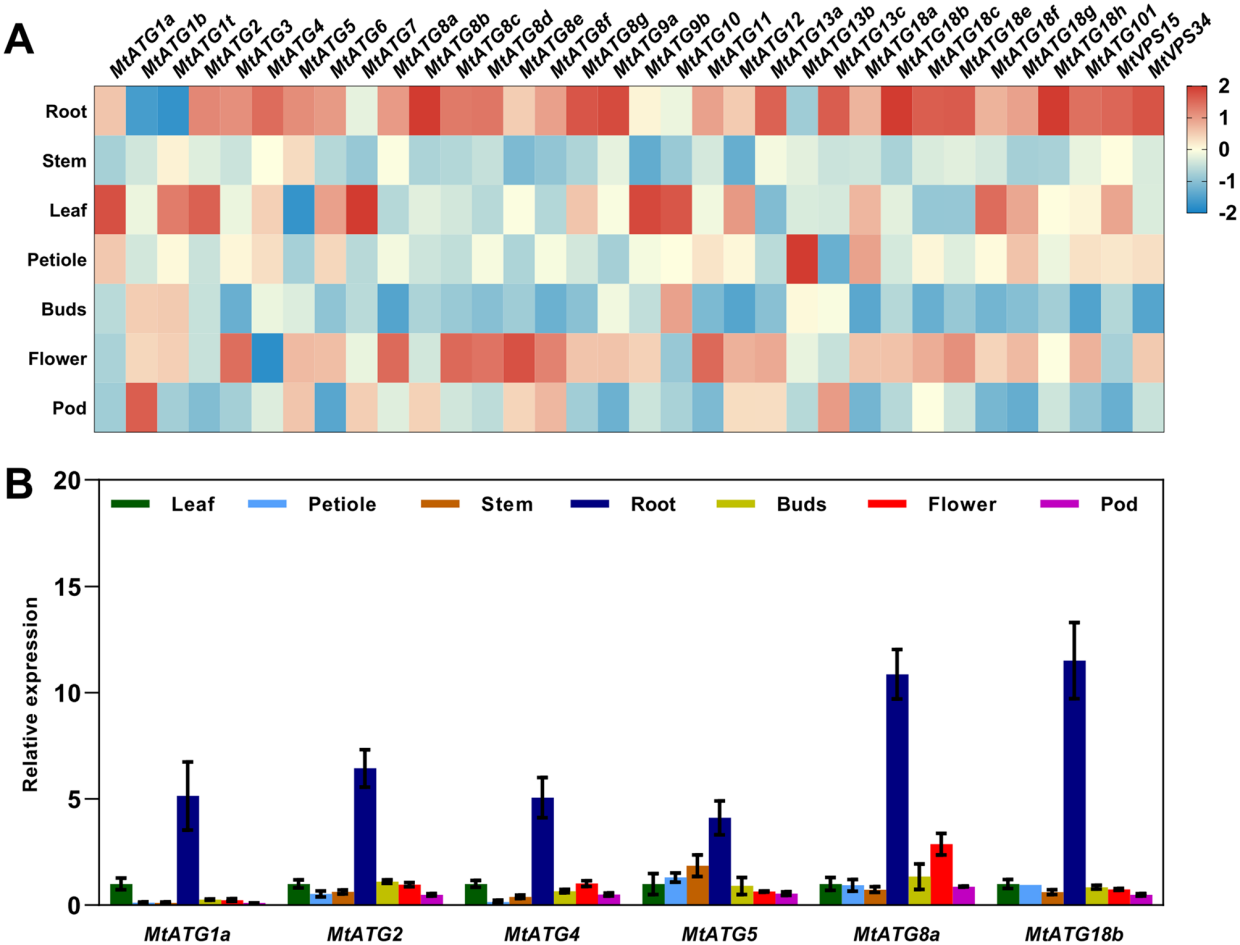
Expression patterns of *MtATGs* in different tissues. (**A**) Expression patterns of *MtATGs* in different tissues. Roots, stems, leaves, petioles, and shoot buds were harvested from multiple *M. truncatula* plants at 28 days after planting, and fully opened flowers and pods (2.5–9.0 mm in length) were collected. (**B**) qRT-PCR validation of *MtATGs* expression in different tissues. *MtACTIN* was used as a reference gene. Error bars represent SD of three independent experiments. Significant differences were indicated with an asterisks (*), P < 0.05.

To investigate the possible roles of *Mt*ATGs in the growth and development of plants, the expression patterns of their genes in different tissues and during different stages of seed development were determined^34^. All the *MtATGs* were expressed in the tested tissues, indicating that autophagy is critical for growth and development of plants (Fig. 6A). However, *MtATGs* showed significantly distinct tissue-specific expression patterns in different tissues. Specifically, the expression levels of many *MtATGs*, such as *MtATG4*, *MtATG8b*, *MtATG8g*, *MtATG9a*, *MtATG13a*, *MtATG13c*, *MtATG18b*, *MtATG18c*, *MtATG18e*, *MtATG18h*, *MtATG101*, *VPS15*, and *VPS34*, were significantly higher in roots than in other tissues (Fig. 6A). In addition, some *MtATGs* (*MtATG1a*, *MtATG1t*, *MtATG2*, *MtATG7*, *MtATG9b*, *MtATG10*, and *MtATG18f*) were highly expressed in leaves, whereas others (*MtATG3*, *MtATG8a*, *MtATG8e*, *MtATG8f*, and *MtATG11*) were highly expressed in flowers (Fig. 6A). The results revealed that different *MtATGs* might function in different tissues. To validate the results of the microarray data, the expressional profiles of several *MtATGs* (*MtATG1a*, *MtATG2*, *MtATG4*, *MtATG5*, *MtATG8a*, and *MtATG18b*) were inspected by qPCR. Most of the selected genes were highly expressed in roots, which was very similar to those of microarray analysis (Fig. 6B).

**Figure 7:**
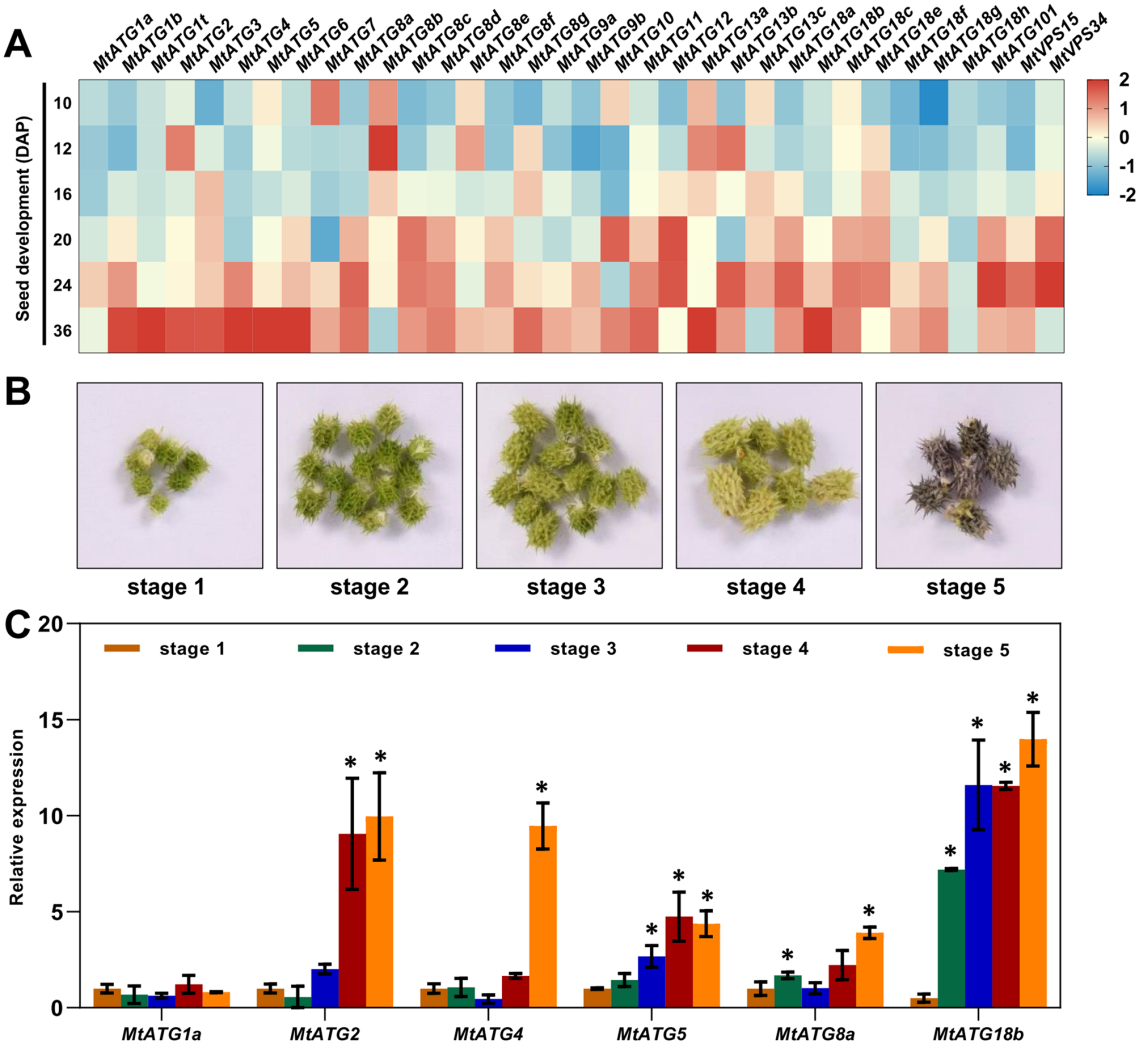
Expression patterns of *MtATGs* during seed development. (**A**) Gene expression of *MtATGs* from microarray data during seed development. Seeds were excised form pods at 10, 12, 16, 20, 24, and 36 days after pollination (DAP). Scale bar represents the relative expression value after z-score normalization. (**B**) Different stages of seed development stages in *M. truncatula*. (**C**) qRT-PCR validation of *MtATGs* expression during seed development. *MtACTIN* was used as a reference gene. Error bars represent SD of three independent experiments. Significant differences were indicated with an asterisks (*), P < 0.05.

Consistent with previous studies, most of the *MtATGs* were upregulated during seed development (Fig. 7A). In particular, *MtATG2*, *MtATG3*, *MtATG4*, *MtATG5*, *MtATG6*, *MtATG13a*, and *MtATG18b*, were highly expressed in the late stage of seed development (Fig. 7A). In contrast, a few *MtATGs*, including *MtATG7* and *MtATG8b*, were downregulated after pollination (Fig. 7A). To validate the results of the microarray data, seeds were collected from pods at 5 different stages of seed developmental (Fig. 7B). As shown in Fig. 7C, the expression levels of five selected genes (*MtATG2*, *MtATG4*, *MtATG5*, *MtATG8a*, and *MtATG18b*) were considerably increased, only *MtATG4* showed no gene expression change during seed development. These results were very similar to those of microarray analysis, and indicate that autophagy is essential for seed development in *M. truncatula*.

### Expression of *MtATGs* in response to drought stress

**Figure 8:**
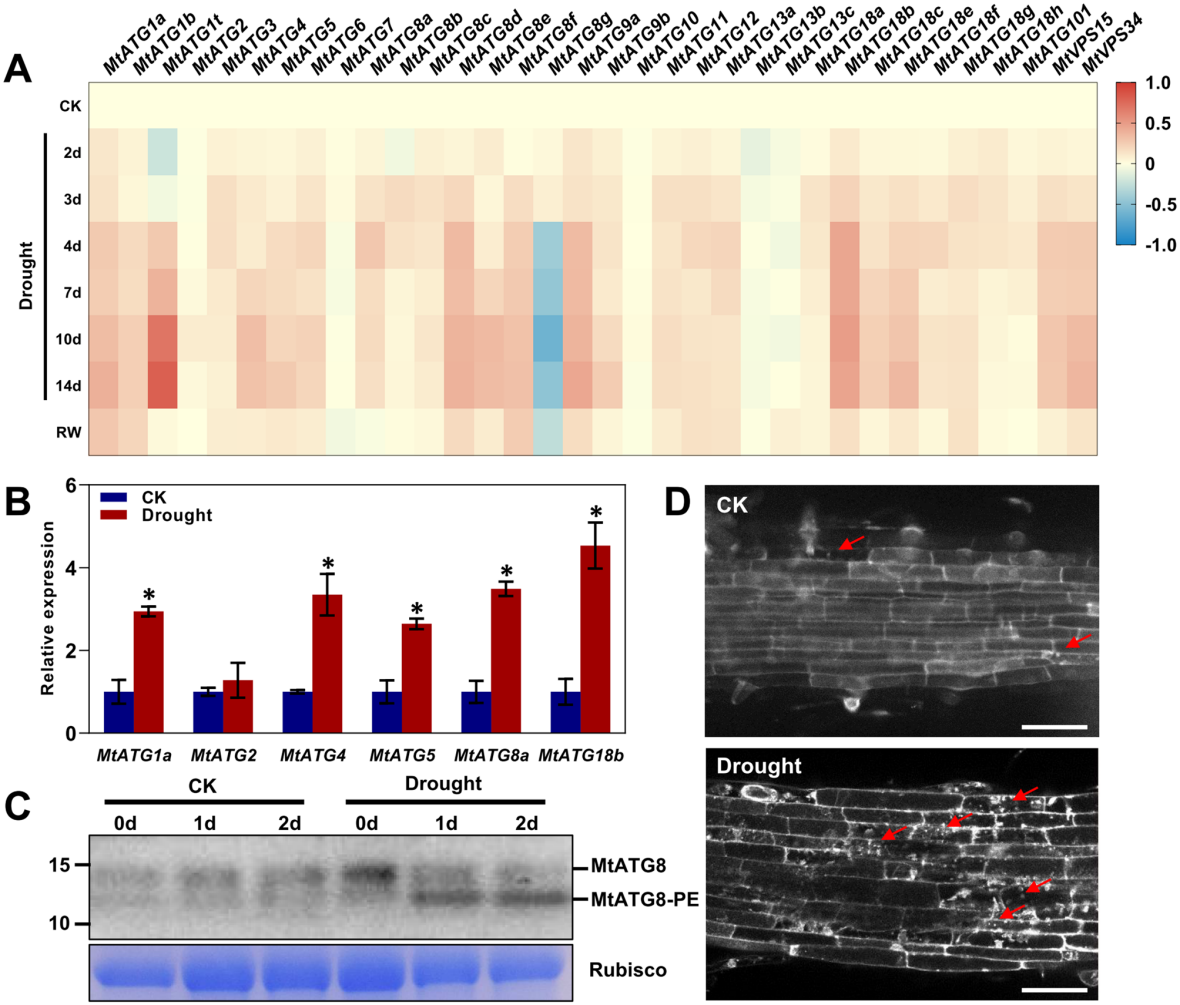
The expression levels of *MtATGs* under drought stress. (**A**) Gene expression of *MtATGs* from microarray data under drought stress. For drought stress treatment, soil-grown plants were subjected to drought stress by withholding watering (Drought) for 14 days, followed by rewatering. Scale bar represents the fold change (log2 value) relative to the corresponding control. (**B**) qRT-PCR validation of *MtATGs* expression under drought stress. For drought stress treatment, 7-day-old seedlings were subjected to drought stress by withholding watering for 2 days. *MtACTIN* was used as a reference gene. Error bars represent SD of three independent experiments. Significant differences were indicated with an asterisks (*), P < 0.05. (**C**) Analysis of ATG8 lipidation by western blot. Two week seedlings were transferred to liquid 1/2 MS medium with or without 300 mM mannitol, and whole seedlings were collected at 0, 1, and 2 day after treatment. The anti-ATG8a antibodies were used for immunoblotting. (**D**) MDC staining of root cells with or without drought treatment. Two-wk-old seedlings were transferred to liquid 1/2 MS medium with or without 300 mM mannitol for 2 days followed by staining with MDC. The labeled autophagosomes (arrows) were visualized by epifluorescence microscopy. Scale bar: 50 μm.

To investigate the putative roles of autophagy in the response of *M. truncatula* to drought stress, the expression profiles of *MtATGs* were analyzed using microarray data from the MtGEA database^35,36^. Generally, most *MtATGs* were upregulated after drought treatment (Fig. 8A). Specifically, 26 of 34 *MtATGs* (e.g., *MtATG1t*, *MtATG8d*, *MtATG9a*, and *MtATG18b*) were continuously upregulated when plants were subjected to drought stress by withholding watering, and the transcripts of *MtATGs* rapidly dropped to their basal levels after resuming the watering (Fig. 8A). Interestingly, *MtATG8g* showed an opposite trend: the expression level of *MtATG8g* dramatically decreased under drought stress compared with other *MtATGs* (Fig. 8A). To validate the results of the microarray data, six genes (*MtATG1a*, *MtATG2*, *MtATG4*, *MtATG5*, *MtATG8a*, and *MtATG18b*) were selected for independent validation by qPCR. The expression levels of most of the selected genes were significantly higher after 2 days of drought treatment (Fig. 8B). To examine autophagy activity under drought stress, antibodies against ATG8a were used to detect ATG8 protein by western blotting. ATG8 proteins are lipidated with phosphatidylethanolamine (PE) to promote autophagosome formation in response to drought treatment, whereas no changes in the level of ATG8-PE were detected under control condition (Fig. 8C). Furthermore, MDC staining showed that the number of autophagosomes was significantly increased after drought treatment (Fig. 8D). These results suggested that autophagy might play a crucial role in *M. truncatula* response to drought stress.

## Discussion

In this study, 39 ATGs were identified in *M. truncatula*. These *ATGs* are similar to orthologous genes in *Arabidopsis*. For example, phylogenetic analysis revealed that *ATG* families in *M. truncatula* are very similar to those in *Arabidopsis*. In addition, the PPI network analysis shows that the interaction pattern of *Mt*ATGs is also similar to that of ATGs in *Arabidopsis.* These results indicate that the autophagy pathway is highly conserved across different plant species. However, the number of members in some ATG families differs among plant species. For example, the ATG8 family contains eight genes in *M. truncatula*, but nine in *Arabidopsis*, seven in rice, and thirteen in wheat^6,9,37^. In addition, the gene structure and conserved domains of some *Mt*ATG families, such as *Mt*ATG16 and *Mt*ATG18 subfamilies, also differ from those of other plants. Furthermore, different types of cis-elements were identified in the promoters of *MtATGs* in the same gene family. These results suggest that *M. truncatula* may have species-specific autophagy mechanism. Hence, it is necessary to illustrate the conserved and specific functions of *Mt*ATGs in future studies.

Autophagy has been shown to play crucial roles in the growth and development of plants^4^. In this study, we found that all *ATGs* were expressed in the tested tissues of *M. truncatula*, but their expression levels varied among different tissues. The tissue-specific expression of *MtATGs* suggests that different functions are required in different tissues. Seed development consists of embryo morphogenesis and seed maturation^38^. In rice, autophagy has been shown to be involved in the regulation of starch and sugar metabolism during seed maturation^39^. In Norway spruce (*Picea abies*), autophagy is also involved in embryogenesis in which it regulates vacuolar cell death of the embryo suspensor^40^. Furthermore, autophagy plays an important role in microspore embryogenesis in *Brassica napus*^41^. The seed weight in autophagy-defective mutants of *Arabidopsis* and maize was reported to be lower than in the wild-type plants^7,42^. In the present study, we found that most of the *MtATGs* were induced during seed development and were highly expressed at the late stage of seed development, which indicates that autophagy is necessary for seed development in *M. truncatula*. Overall, autophagy plays crucial roles in the growth and development of plants through a pathway that is conserved across different species.

Autophagy has been demonstrated to promote plant survival by maintaining cellular homeostasis under drought stress^43,44^. In *A. thaliana*, the transcriptional level of *ATG18a* was rapidly upregulated by mannitol treatment^45^. In *O. sativa*, the expression levels of *OsATG6* genes were also induced by drought stress^46^. Moreover, ATG genes were upregulated by drought stress in many other plant species, such as barley^47^, pepper^48^, apple^49^, and banana^50^. Besides changes in gene expression, the *Arabidopsis* autophagy-defective mutants (*atg5*, *atg7*, and *RNAi-ATG18a*) showed more sensitivity to drought treatment than the wild type^45,51^. Inhibition of autophagy by 3-MA or knockdown of *ATG6* sensitized wheat seedlings to drought stress^52^. Furthermore, virus-induced gene silencing of *ATG8d* or *ATG18h* significantly reduced drought tolerance in tomato^53^. However, overexpression of *MdATG5* or *MdATG18a* enhanced tolerance to drought stress in apple trees^54,55^. In addition, overexpression of *SiATG8a* from foxtail millet improved drought tolerance in *Arabidopsis*^56^. Recently, it was reported that autophagy improves drought tolerance in *M. truncatula* through degradation of the aquaporin MtPIP2;7, which interacts with the cargo receptor MtCAS31^56^. Consistent with previous studies, our results reveal that the promoter of many *MtATGs* contain the drought-related MBS cis-element. Furthermore, the transcriptional levels of most of the *MtATG* genes, especially those of the *MtATG8* family, significantly increased after drought treatment. The lipidation of ATG8 protein and accumulation of autophagosome are enhanced in *M. truncatula* during drought stress. Our findings indicate that autophagy is is largely induced by drought stress in *M. truncatula*, and can be considered an adaptive response under drought stress.

## Conclusion

This study provided comprehensive analysis of *ATGs* in *M. truncatula*. In total, 39 *ATGs* were identified in *M. truncatula*. Members of the same *ATG* family showed similar gene structure and conserved domains. Analysis of cis-elements implied that *MtATGs* have potential biological functions in response to phytohormone, abiotic and biotic stresses. Phylogenetic and interaction network analyses suggested that the function of *Mt*ATGs is evolutionarily conserved in *Arabidopsis* and *M. truncatula*. The expression pattern of *MtATGs* indicates that autophagy possibly participates in seed development and plays an important role in plant responses to drought stress. In conclusion, this study gives a detailed overview of *MtATGs* and their expression patterns. The results obtained in this study provide useful information for further functional characterization of autophagy in *M. truncatula*.

## Supplementary Information

Supplementary Figures.

Supplementary Table S1.

Supplementary Table S2.

Supplementary Table S3.

Supplementary Table S4.
