# Supplementary material for: Genome-wide analysis of autophagy-related genes in Medicago truncatula highlights their roles in seed development and response to drought stress
Source: Sci Rep. 2021 Nov 25;11:22933. doi: 10.1038/s41598-021-02239-6 (PMC8616919; doi:10.1038/s41598-021-02239-6)
Supplement: Supplementary file 1 — Supplementary Figures. [file 41598_2021_2239_MOESM1_ESM.docx]

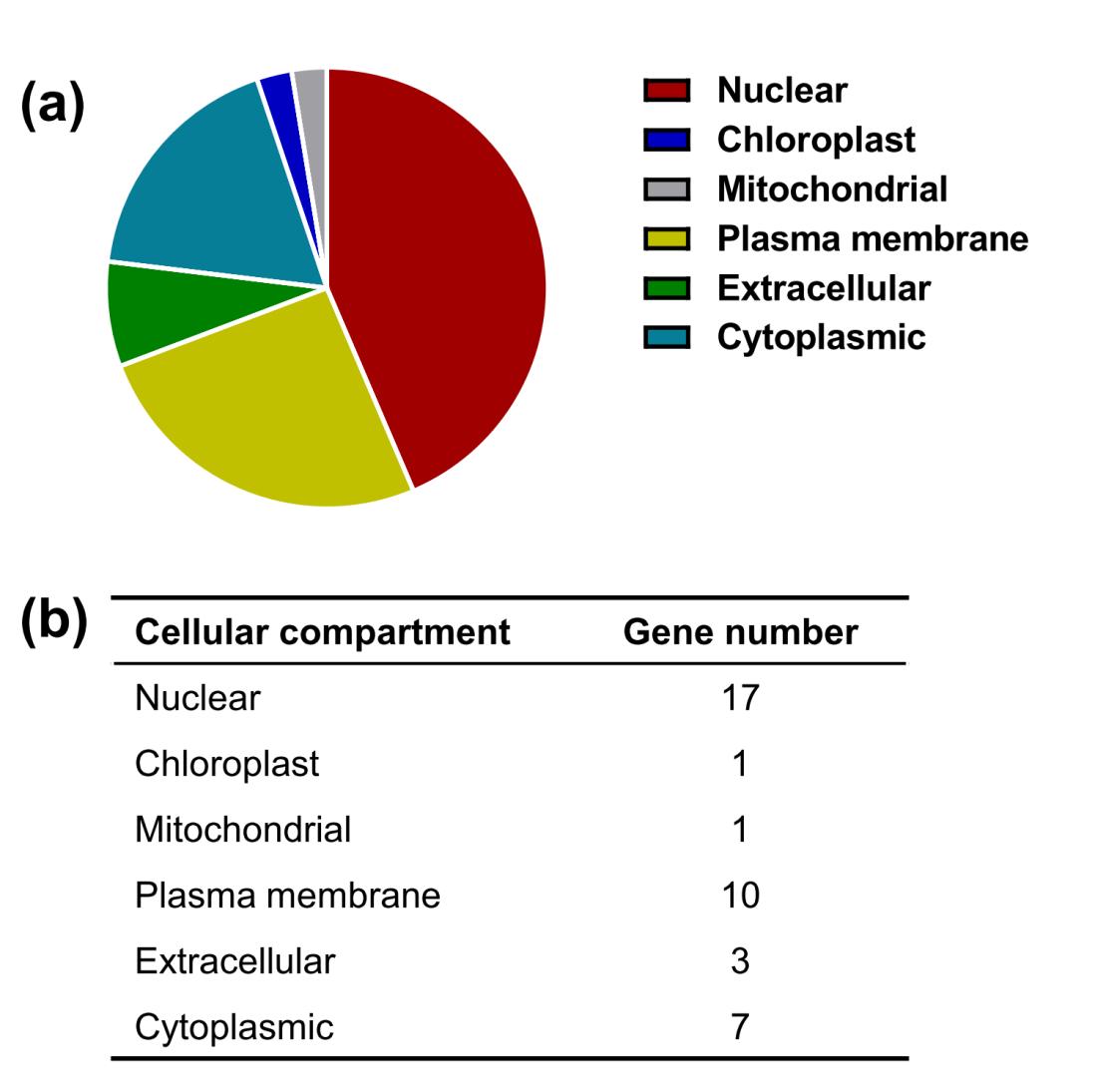


**Supplementary Figure S1. The distribution of subcellular localization of ATG proteins in *Medicago truncatula*.**

(A) Locations of ATGs predicted by the CELLO system. (B) Number of ATGs assigned to each cellular compartment.


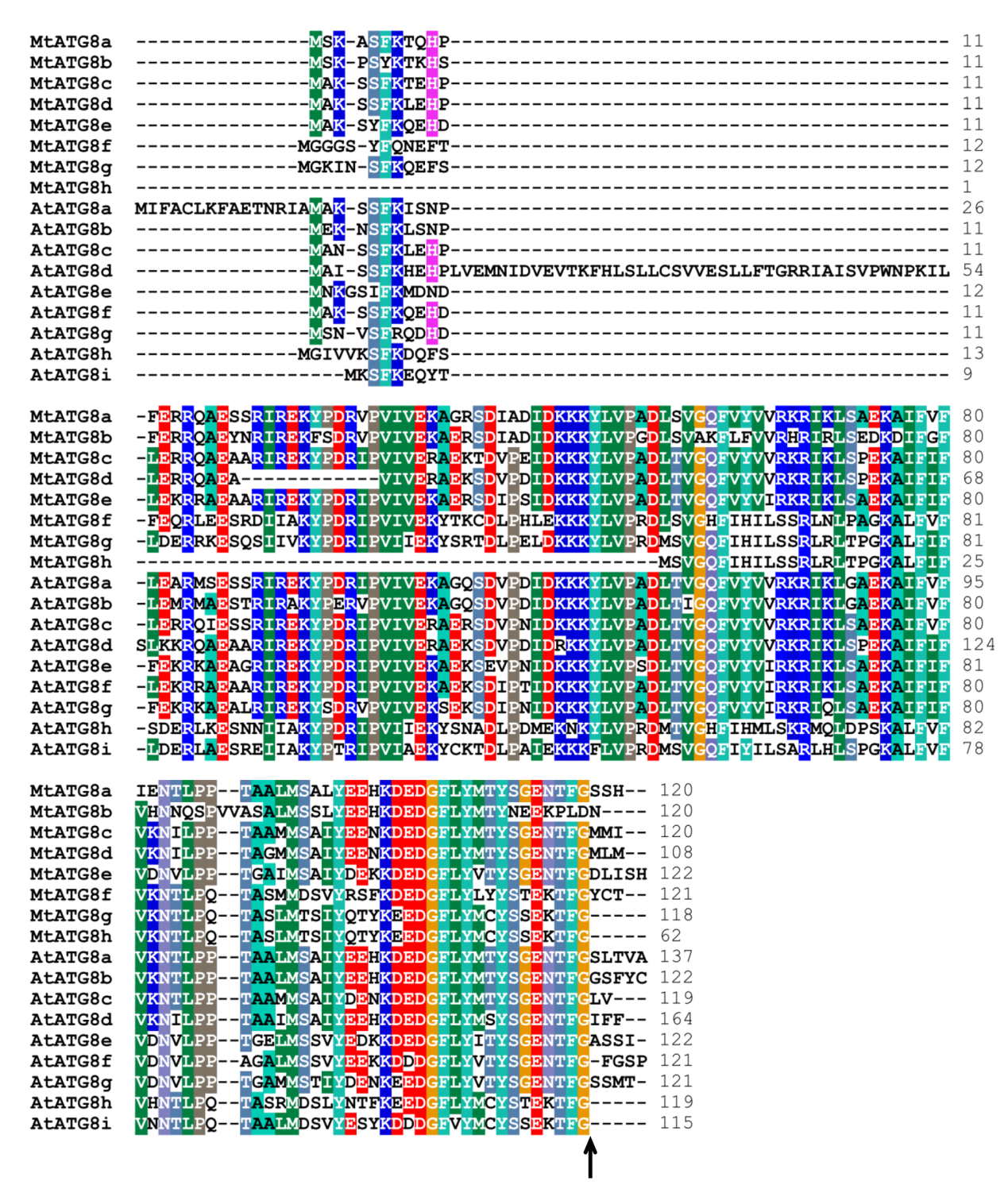


**Supplementary Figure S2. Protein alignment of the ATG8 family from *Medicago truncatula* and *Arabidopsis thaliana*.**

The full amino acid sequences of *Mt*ATG8s and *At*ATG8s were aligned using the ClustalW algorithm. The scissile ATG4 cleavage site is shown by the vertical black arrow.


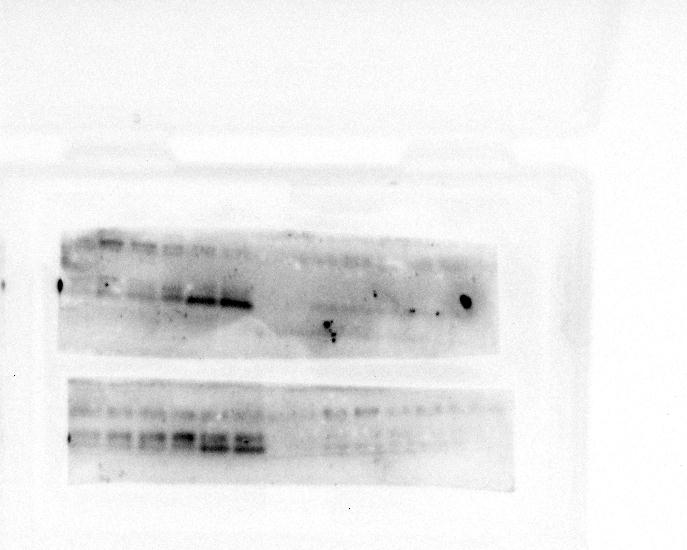


**Supplementary Figure S3. Full length blots for Figure 8C.**

Membranes of transfers from 15% gels were routinely cut between the 10 kD and 25 kD proteins prior to Anti-ATG8a antibodies incubations. The immunoblots were repeated twice with independent biological samples.


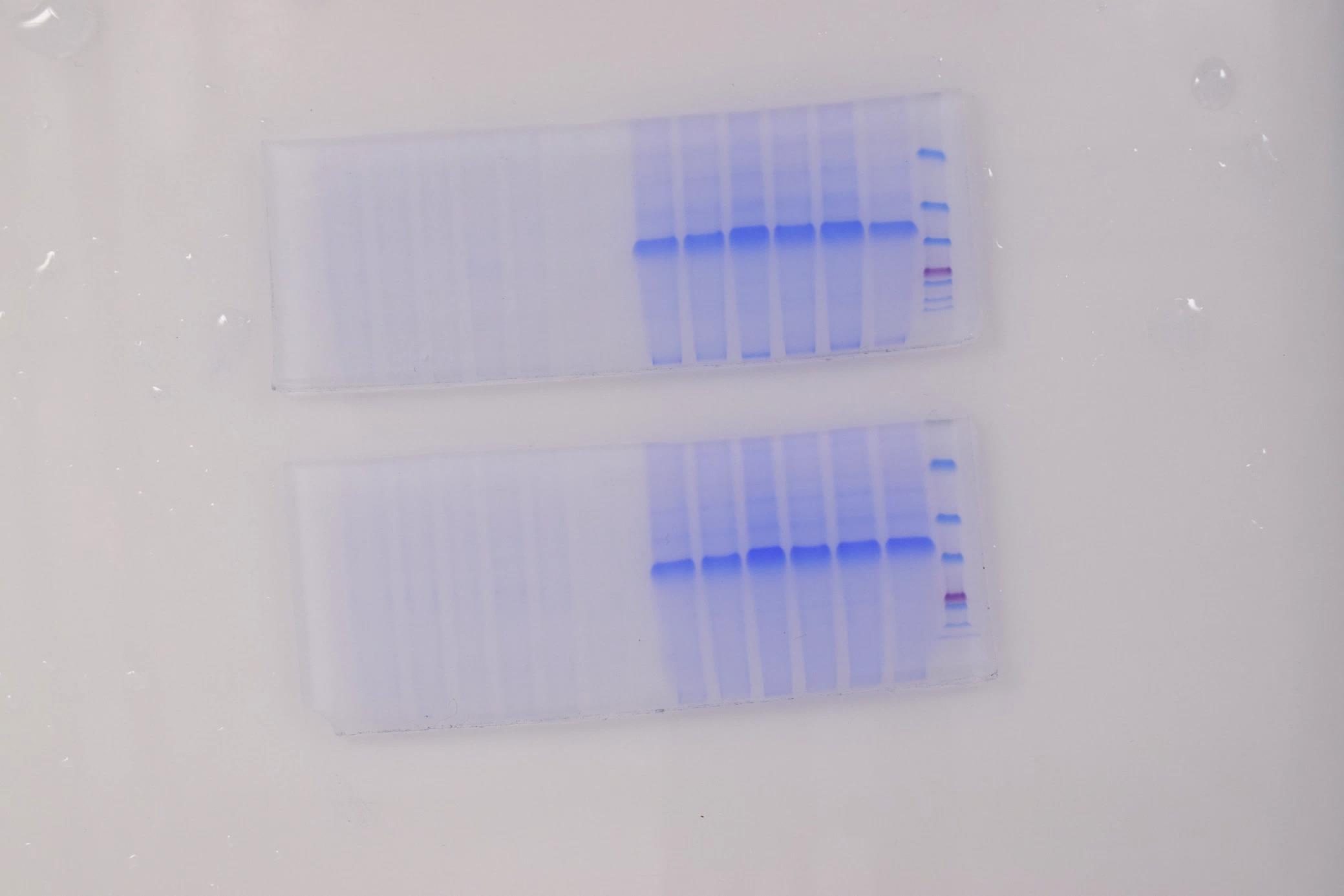


**Supplementary Figure S4. Full length gels for Figure 8C.**
