# Supplementary material for: Genome-wide analysis of autophagy-related genes in Medicago truncatula highlights their roles in seed development and response to drought stress
Source: Sci Rep. 2021 Nov 25;11:22933. doi: 10.1038/s41598-021-02239-6 (PMC8616919; doi:10.1038/s41598-021-02239-6)
Supplement: Supplementary file 5 — Supplementary Table S4. [file 41598_2021_2239_MOESM5_ESM.docx]

**Supplementary Table S4.** Sequences of primers used in qPCR.

| **Gene name** | **Direction** | **Sequence (5’→3’)** |
| --- | --- | --- |
| *MtACTIN* | Forward | GCAGATGCTGAGGATATTCAACC |
|  | Reverse | CTTCGTCACCAACATAGGCATCC |
| *MtATG1a* | Forward | GCTCTAGACATCTGTAGCACTT |
|  | Reverse | TAGTTTCTCAGCACGATTGACT |
| *MtATG2* | Forward | TGAAGGTGTGAAAACTATTGCG |
|  | Reverse | AAACACGAAGAACCAAAGTACG |
| *MtATG4* | Forward | TTAGAGTTTTCAAGGGGCCTAG |
|  | Reverse | CCCAGTAATATTAACAACCGGC |
| *MtATG5* | Forward | ACCACACTTCCTCCTGGAGT |
|  | Reverse | TCACCGTCACATGGAAGCAA |
| *MtATG8a* | Forward | CTGCTCTGATGTCTGCCCTT |
|  | Reverse | GTGGGAGGAACCAAAGGTGT |
| *MtATG18b* | Forward | AGTCTTCTTGCTATAGTCGGTG |
|  | Reverse | GAGTCTTTTTCGATTCATGCGA |
